# Lung neoplasm mimicking as ectopic pregnancy due to paraneoplastic secretion of human chorionic gonadotropin: a case report and literature review

**DOI:** 10.1007/s00404-020-05927-2

**Published:** 2021-01-04

**Authors:** Jin Peng, Shangge lv, Lin Liu, Shuai Feng, Naidong Xing

**Affiliations:** 1 Department of Obstetrics and Gynecology, Qilu Hospital, Shandong University, Jinan, 250012 Shandong People’s Republic of China; 2 School of Medicine, Institute of Diagnostics, Shandong University, Jinan, 250012 Shandong People’s Republic of China; 3 Department of Pathology, Qilu Hospital, Shandong University, Jinan, 250012 Shandong People’s Republic of China; 4 Shandong Cancer Hospital and Institute, Shandong First Medical University and Shandong Academy of Medical Sciences, Jinan, 250117 Shandong People’s Republic of China; 5 Department of Urology, Qilu Hospital, Shandong University, Jinan, 250012 Shandong People’s Republic of China

**Keywords:** Beta-human chorionic gonadotropin, β-HCG, Lung neoplasm

## Abstract

**Purpose:**

The present systematic review aimed to examine the relationship between lung neoplasm and human chorionic gonadotropin (HCG). Especially, women with lung neoplasm mimicking as ectopic pregnancy were explored.

**Methods:**

A rare case of lung neoplasm with high serum β-HCG, which was initially thought to be ectopic pregnancy, was reported. A literature search was performed of the US National Library of Medicine (MEDLINE), EMBASE, PubMed, and the Cochrane Database of Systematic Reviews using appropriate keywords and subject headings to February 2020.

**Results:**

Studies assessed lung neoplasm patients with positive HCG were included. Twenty studies, including 24 patients, were included. These cases illustrate the importance of considering the possibility of paraneoplastic secretion of β-HCG in patients who have a positive pregnancy test. This may prevent a delay in the diagnosis and treatment of malignancy in young women. Of the 24 cases, only 7 (29.17%) were managed surgically; others were managed conservatively or with chemotherapy or radiation.

**Conclusion:**

The present systematic review shows the need to re-awaken awareness and high index of suspicion to lung neoplasm diagnosis in patients with positive pregnancy test.

## Introduction

Beta-human chorionic gonadotropin (β-HCG) belongs to the glycoprotein hormone family and is usually used to exclude pregnancy for patients. Human chorionic gonadotropin (HCG), a 38-kDa glycoprotein hormone, is physiologically produced by syncytiotrophoblast cells in the placenta during pregnancy. It comprises two subunits (α and β polypeptide chains) which are joined by noncovalent bonds. Since the α-subunit of HCG has a sequence homology similar to other pituitary hormones, including luteinising hormone (LH), thyroid-stimulating hormone (TSH) and follicle-stimulating hormone (FSH), the β-subunit of HCG is assayed. HCG is a specific marker for trophoblastic tumors of placenta and gestational tumors [1]. Typically, serum β-HCG levels are markedly raised in germ cell tumors, particularly pure choriocarcinoma [2]. Ectopic secretion of β-HCG by other tumors is rare. Ectopic expression of β-HCG was found in epithelial carcinoma, especially for tumors of the stomach, endometrium, ovary, cervix and lung [3–8]. Paraneoplastic syndromes associated with ectopic β-HCG production have been previously described in oropharyngeal squamous cell carcinoma, urothelial carcinoma of bladder, leiomyosarcoma and breast [9–12]. To date, only fewer β-HCG-secreting lung neoplasm have been reported in the literature. A young woman presenting amenorrhea symptoms with high serum β-HCG was initially thought to be ectopic pregnancy at our department. Because this case is rare, we here report it and review the literature.

## Methods

**Fig. 1:**
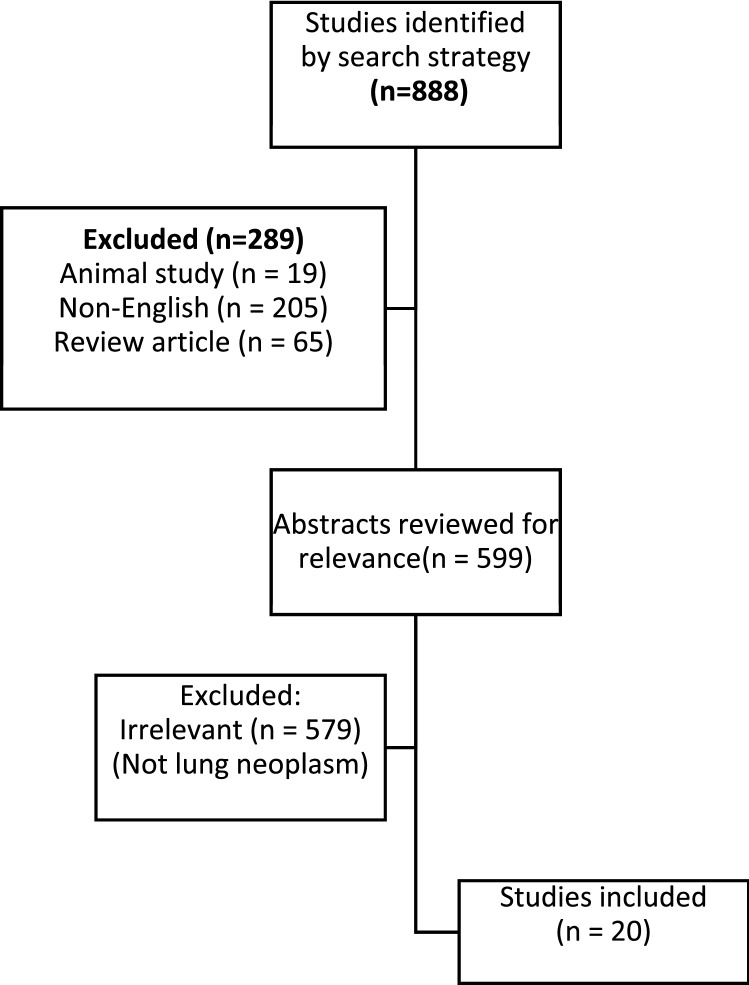
Flowchart demonstrating study selection

A systematic literature search of the US National Library of Medicine (MEDLINE), the Excerpta Medical Database (EMBASE), PubMed, and the Cochrane Database of Systemic Reviews (CDSR) was made using the following search criteria: ‘‘lung neoplasm’’ AND (beta-human chorionic gonadotropin OR HCG). Included data were from the inception of the searched databases until Feb. 2020. The selection process is summarized in Fig. 1. Articles were excluded if they were animal studies, not in the English language, were review articles, were not related to lung neoplasm or did not use either HCG OR beta-human chorionic gonadotropin. Table 1 summarizes the included studies for lung neoplasm and HCG analyzed.

**Table 1:** Summary of reported cases of lung neoplasm with secretion of HCG

Paper no	Author	Year	Country	Number of cases	Age (years)	Sex	Presentation	HCG/β-HCG	Treatment	Pathology
1	Noda Y et al. [17]	1990	Japan	2	66/65	Male	Bilateral gynecomastia	Elevated	Chemotherapy	Large cell carcinoma
2	Smith LG et al. [18]	1992	USA	1	Unknown	Female	Unknown	Elevated	Unknown	Adenocarcinoma
3	Liu MT et al. [19]	1993	China	1	Unknown	Male	Bilateral gynecomastia dyspnea	> 2000	None	Adenocarcinoma
4	Arano Y et al. [20]	1994	Japan	1	72	Female	Cough	Elevated	Surgery	Adenocarcinoma
5	Snyder RW et al. [14]	1995	Canada	1	31	Female	Abdominal pain amenorrhea	44	Chemotherapy	Adenocarcinoma
6	Uckaya G et al. [21]	1998	Turkey	1	50	Male	Unknown	elevated	Unknown	Adenocarcinoma
7	Yoshida J et al. [22]	2000	Japan	1	31	Female	Amenorrhea	12,238	Surgery	Squamous cell carcinoma
8	Sawa H et al. [23]	2001	Japan	1	58	Male	Pain	Elevated	Surgery	Adenocarcinoma
9	Sagaster P et al. [13]	2002	Austria	1	30	Female	Abdominal pain, amenorrhea	449	Surgery	Adenocarcinoma
10	Mehta H et al. [24]	2008	USA	1	37	Female	Cough dyspnea	10,273	Surgery	Large cell carcinoma
11	Okutur K et al. [16]	2010	Turkey	1	50	Female	Bilateral gynecomastia weight loss	6500	Surgery and chemotherapy	NSCLC
12	Khobta N et al. [25]	2012	France	2	45/64	Female	Unknown	19/13	Chemotherapy and targeted therapy	NSCLC/adenocarcinoma
13	Attia S et al. [26]	2012	Israel	1	47	Female	Unknown	Elevated	Unknown	Squamous cell carcinoma
14	Brandon C et al. [15]	2012	USA	1	46	Female	Left thigh pain	308	Radiation and chemotherapy	Adenocarcinoma
15	Vicier C et al. [27]	2013	France	1	43	Female	Amenorrhea chest pain	3393	Chemotherapy	Adenocarcinoma
16	Godbert et al. [28]	2013	France	2	unknown	Female/male	Unknown	Elevated	Chemotherapy	NSCLC
17	Wong Y et al. [29]	2015	Malaysia	1	62	Female	Dyspnea weight loss	11,212	Chemotherapy	Adenocarcinoma
18	Cirit KB et al. [30]	2016	Turkey	1	43	Male	Dyspnea, bilateral gynecomastia	4261	Chemotherapy	NSCLC
19	Groza D et al. [31]	2017	Switzerland	1	48	Female	None	Elevated	Chemotherapy	NSCLC
20	Khattri S et al. [32]	2011	USA	1	68	Male	Weight loss	11,286	None	NSCLC
	Index	2017	China	1	28	Female	Amenorrhea	54	Surgery	Adenocarcinoma

*NSCLC* non-small cell lung cancer, *unknown* unconfirmed data; HCG/β-HCG(mIU/mL)

## Case report

**Fig. 2:**
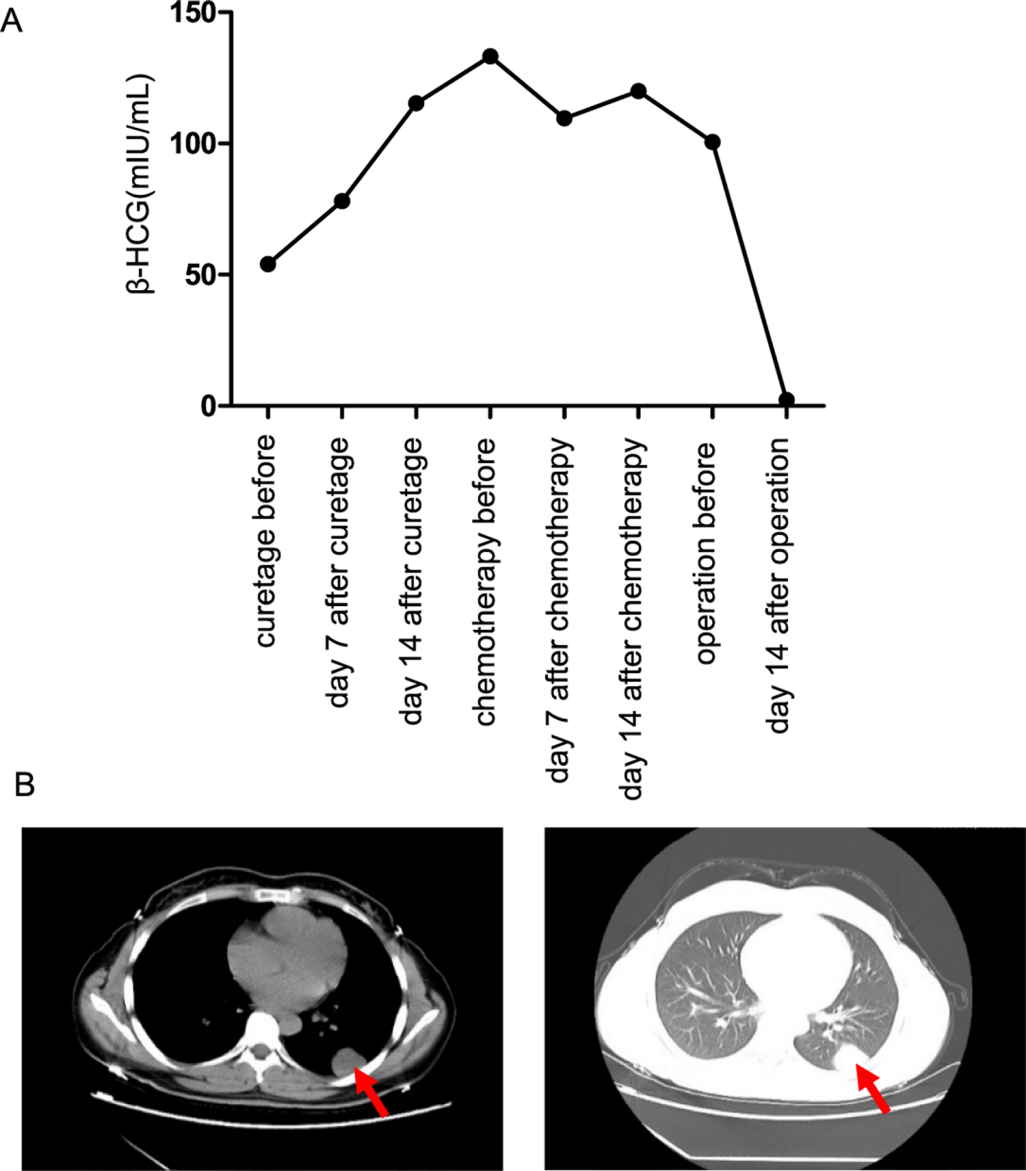
**a** Serum β-hCG showed before and after the treatment of curettage, chemotherapy and operation. **b** Computed topography (CT) abdomen and thorax shows a left lower lobe mass (long arrow) which displaces

**Fig. 3:**
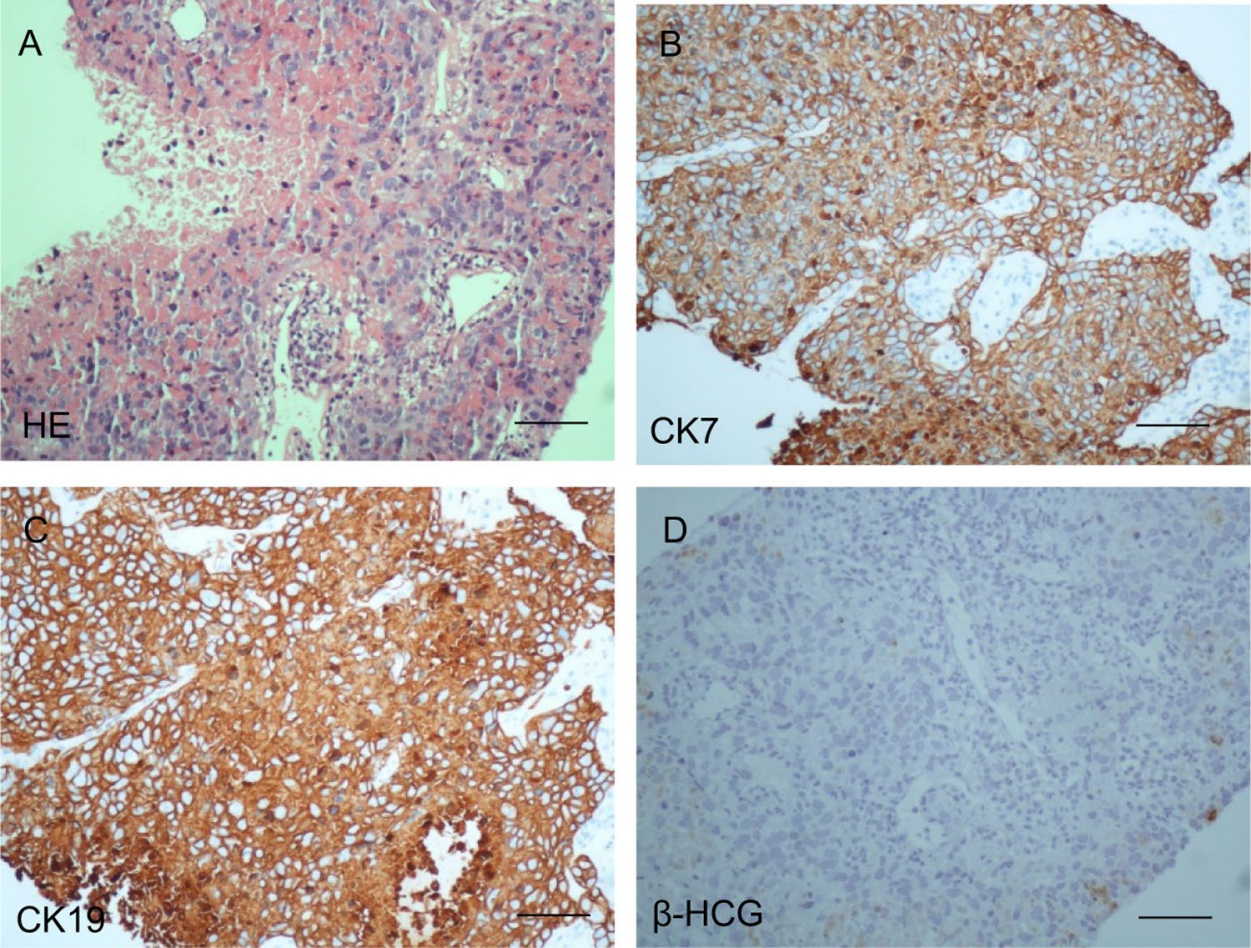
Histological and immunohistochemistry features of lung neoplasm; **a** HE of malignant cells; labelling for **b** CK7 and **c** CK19; **d** β-hCG

A 28-year-old female, a non-smoker, was presenting amenorrhea symptoms, who delivered a baby four years ago and had partial hydatidiform mole history two years ago. Serum β-HCG levels were found to be high, measuring 54 mIU/ml (normal upper limit 10 mIU/ml in non-pregnant woman), which prompted the initial suspicion of pregnancy and further investigations to exclude an ectopic pregnancy or choriocarcinoma. Abdominal and pelvic ultrasonography showed no obvious gestation sac but 1-cm low echo mass. Then, curettage of uterine cavity was performed but no pregnant chorionic villi tissue found by histopathological examination, also β-HCG elevated as 78 mIU/ml at 7th day after curettage. Serum β-HCG persisted increasingly is 115.3 mIU/ml at 14th day after curettage. As suspected diagnosis of ectopic pregnancy, the patient was treated with mifepristone for 3 days and started on chemotherapy with MTX for 7 days. Biochemical response was observed after the first cycle of chemotherapy with downregulation in serum β-HCG levels from 133.2 to 109.5 mIU/ml. However, Serum β-HCG is still 119.9 mIU/ml at 14th day after chemotherapy (Fig. 2a). Meanwhile, abdominal and pelvic ultrasonography yielded no abnormal findings. Incidentally, X-ray check found a 3-cm round-like high density mass in the left lower lobe lung. Furthermore, a computed tomography (CT) scan of the thorax, abdomen and pelvis following an abnormal chest radiograph disclosed a 3-cm left lower lobe lung mass (Fig. 2b). No other focal lesions were detected. So, a CT-guided biopsy of the left lung mass was subsequently performed. Histopathological examination revealed adenocarcinoma. The malignant cells displayed moderate nuclear pleomorphism, vesicular nuclei with prominent nucleoli and abundant vacuolated to eosinophilic cytoplasm. Atypical mitosis was occasionally encountered (Fig. 3a). There were no multinucleated giant cells, syncytiotrophoblasts and cytotrophoblasts to suggest choriocarcinoma. Immunohistochemically, the malignant cells were diffusely positive for cytokeratin 7 (CK7) and cytokeratin 19 (CK19) (Fig. 3b–c). Other immunomarkers, i.e., cytokeratin 5/6 (CK5/6), TTF-1 and napsin A were negative. β-HCG is negative, but some of the malignant cells also displayed focal immunopositivity for β-HCG (Fig. 3d). A histological diagnosis of β-HCG-secreting low differentiated adenocarcinoma of the lung was rendered. Then, the patient and family opted for the left lower lobe resection and upper lobe tumor wedge resection. The preoperative and 2-week postoperative HCG levels were 100.5 and 2.3mIU/ml, respectively (Fig. 2a). So far, the patient has survived more than 28 months after operation.

## Results

**Fig. 4:**
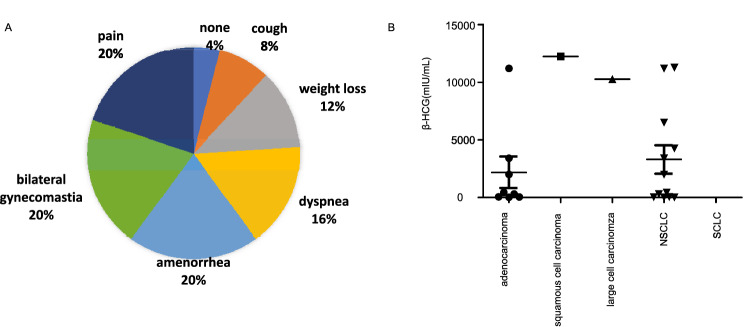
The presentation (**a**) and histological pathology types (**b**) of reported cases of lung neoplasm with secretion of HCG

A total of 24 cases of lung neoplasm with elevated beta-human chorionic gonadotropin including index one were identified (Table 1). Excluding unfirmed data, it consists of 7 male and 17 female patients with elevated mean level of HCG which is 6703 mIU/mL. Mean age of these cases is 49.8 years. The presentation of 24 patients shows diversity, 8% cough, 20% pain,12% weight loss,16% dyspnea and 40% ectopic pregnancy-related symptoms (20% amenorrhea and 20% bilateral gynecomastia), while 4% patients have no presentation (Fig. 4a). Only 7 patients of them have chance to receive operation and 14 patients received chemotherapy and/or radiation and/or targeted therapy. 2 patients died soon after diagnosis who do not receive treatment and 2 cases revealed without treatment data. The histological type of these cases is 7 non-small cell lung cancer (NSCLC), 2 squamous cell carcinomas, 12 adenocarcinoma and 3 large cell lung cancer (Fig. 4b). Interestingly, we did not find the histological type of small cell lung cancer (SCLC) case. The elevated mean HCG level of adenocarcinoma and NSCLS is 2184 mIU/mL and 5516 mIU/mL, respectively.

## Discussion

We report a case of a young female patient who presented with amenorrhea symptoms and was found to have a positive pregnancy test. Subsequently, she was found to have lung adenocarcinoma with ectopic β-HCG secretion. Ectopic production of a variety of hormones such as ADH, parathyroid hormone, ACTH, insulin-like growth factor, thus leading to presentation as endocrine syndromes in patients with lung cancer are well known. Little is known about paraneoplastic secretion of β-HCG. Expression of ectopic β-HCG is not associated with any specific histological subtypes, although some authors have found that this phenomenon is more common in lung adenocarcinoma. Interestingly, according to reported cases, we observed diverse histological pathology cases excluding small cell lung cancer (SCLC), maybe due to limited cases. As the same as most reported cases of β-HCG-secreting lung adenocarcinoma, our report shows that serum β-HCG levels of adenocarcinoma is 2184 mIU/mL. Although no studies showed β-HCG levels appropriate as a diagnostic marker for lung cancer, future studies may benefit from large sample study to assess the comprehensive paraneoplastic β-HCG production by lung carcinoma of various histological types.

The precise mechanism of β-HCG secreted by non-trophoblastic tumors is poorly understood. The ectopic production of trophoblastic hormones by lung carcinoma has not been investigated systemically. Recent findings suggest that glycoprotein- and protein hormones act as local paracrine growth in normal and malignant tissues. Besides their important biological role in promoting progesterone production in the uterine vasculature during pregnancy, free β-HCG was recently found to play a major role in the tumorigenesis of non-trophoblastic tumors [13]. They acted as an autocrine anti-apoptotic and angiogenic growth factor, resulting in cancer cell growth. This may also explain the chemoresistance and aggressiveness of β-HCG-secreting lung cancer. Some authors have shown a high expression of β-HCG in metastatic lung cancer cell lines, which may attribute to the metastatic potential of such secreting tumors [14]. The findings supported the potential use of serum β-HCG to predict patients who were at risk of developing metastatic disease. In our case, immunohistochemistry analysis of lung adenocarcinoma revealed negative staining of cancer cells, since serum β-HCG was low, maybe due to early stage of lung cancer.

At present, no chemotherapy regimen is available for such rare tumors. Interestingly, slight reduction of serum β-HCG level was observed after chemotherapy. Serial measurements of circulating β-HCG seemed to be influenced by anti-neoplastic treatments. The serum β-HCG level returned to normal 2 weeks after the operation.

Lung cancer mortality is strongly associated with the predominant diagnosis of late stage lesions that hampers effective therapy [15]. The level of β-HCG measured in tumor tissues in NSCLC patients was not of prognostic, diagnostic or predictive significance for OS or recurrence after treatment [16]. We identified that the mean age at diagnosis for β-HCG-secreting lung neoplasm was 50 years old, which was much older than the usual age for pregnancy-related disease. Otherwise, our case is 28 years old. We omit X-ray or CT examination of chest. So that our case was misdiagnosed at first. We here firstly revealed that β-HCG-secreting lung neoplasm presented with 40% ectopic pregnancy-related symptoms (20% amenorrhea and 20% bilateral gynecomastia), which was easily confused with extrauterine pregnancy clinically. Only 29.17% (7 out of 24 cases) had chance to receive operation, maybe due to delay diagnosis. Fortunately, our case could be operated on for lung cancer immediately. Thus, here we emphasize the need to aware of such young women cases with elevated HCG which is related not only with pregnancy-related disease, but also the lung cancer.

## Conclusion

In conclusion, the present systematic review shows that β-HCG-secreting lung neoplasm is easily confused with extrauterine pregnancy clinically. Therefore, knowing the expression of β-HCG by lung tumors may facilitate prompt diagnosis and initiation of appropriate intervention in time. This review examines the literature regarding a known indicator β-HCG of lung cancer, maybe it is the first step in exploring further research about the role of β-HCG in lung neoplasm.
